# Correction: Scoping review and interpretation of myofascial pain/fibromyalgia syndrome: An attempt to assemble a medical puzzle

**DOI:** 10.1371/journal.pone.0330858

**Published:** 2025-09-11

**Authors:** The PLOS One Editors

## Notice of Republication

This article [1] was republished on August 5, 2025, to replace Figure 5 due to a permissions issue: the original published version of Figure 5 included content that was published previously by Reuters [2] that is not available under a CC BY license. Please download this article again to view the correct version.
