# Supplementary material for: Scoping review and interpretation of myofascial pain/fibromyalgia syndrome: An attempt to assemble a medical puzzle
Source: PLoS One. 2022 Feb 16;17(2):e0263087. doi: 10.1371/journal.pone.0263087 (PMC8849503; doi:10.1371/journal.pone.0263087)
Supplement: S1 File — (DOCX) [file pone.0263087.s002.docx]

**Supplementary material for "Scoping review and interpretation of Myofascial Pain/Fibromyalgia syndrome: an attempt to assemble a medical puzzle"**

# Methods

# During the process of the review, certain topics rose that not were not sufficiently covered in the literature found through the systematic search. Therefore, further literature (through searches that were not systematic) was gathered for these topics. These topics were: myofibroblasts contractions and generation of tension, neurology of myofascial pain, myofascial pain and movement, fascial properties, searching pubmed for the term "unexplained", etc.

# Using "ovid" engine to search EMBASE yielded 0 results for the phrase "fascia tension pain" (whereas 127 items were found on PUBMED) therefore a broader search was performed in this database using only two word combinations: "fascia tension" (yielded 10 results) and "fascia pain" yielded 15 results (N=25 combined)

# A search on COCHRANE was not done for the phrases "fascia tension pain" and "fascia stiffness pain" as the reviewer sought for controlled trials for myofascial pain syndrome, more than 150 items from COCHRANE oriented for MPS sufficed for this purpose.

# Searches for "sympathetic activity induced by pain" and "Spinal mobilization sympathetic nervous system" was expanded for a search in all fields from inception only in databases where no results were found in title/abstract.

# For "myofascial pain syndrome" the search was done on Cochrane but was limited to the title field only as it was too broad. This yielded 138 results.

# Information gathered was summarized in excel document and a long word document containing key information from items. The word document evolved into the article, over time.

# Needling

To grasp the framework of needling, the reader may try and imagine a geodesic dome connected not by straight solid bars, but imagine it connected by extremely thin sheets of spandex/elastane. Each sheet can change its spring constant. Then imagine continuously applying external forces to this dome from different directions.

# Insertion of needles in many "scattered" points while at rest will allow the system to realign appropriately and accordingly to the inherent internal forces or pullies, e.g., the skeleton. Now the reader may imagine this dome only in the shape of a human, and it can move.
